# Minimally invasive adrenalectomy for large pheochromocytoma: not recommendable yet? Results from a single institution case series

**DOI:** 10.1007/s00423-021-02312-8

**Published:** 2021-09-01

**Authors:** Simone Arolfo, Giuseppe Giraudo, Caterina Franco, Mirko Parasiliti Caprino, Elisabetta Seno, Mario Morino

**Affiliations:** 1 Department of Surgical Sciences, University of Torino, Corso A. M. Dogliotti 14, 10126 Turin, Italy; 2 Department of Medical Sciences, University of Torino, Turin, Italy

**Keywords:** Pheochromocytoma, Minimally invasive surgery, Large adrenal mass, Hypertensive peaks, Postoperative hypotension, Case series

## Abstract

**Background:**

Minimally invasive adrenalectomy represents the treatment of choice of pheochromocytoma (PCC). For large or invasive PCCs, an open approach is currently recommended, in order to ensure complete tumor resection, prevent tumor rupture, avoid local recurrence, and limit perioperative hemodynamic instability. The aim of this study is to analyze perioperative outcomes of laparoscopic adrenalectomies (LAs) for large adrenal PCCs.

**Methods:**

All consecutive LAs for PCC performed at a single institution between 1998 and 2020 were included. Two groups were defined: lesions larger (group 1) and smaller (group 2) than 5 cm. Short-term outcomes were compared in order to find any significant difference between the two groups.

**Outcomes:**

One hundred fourteen patients underwent LA during the study period: 46 for lesions larger and 68 for lesions smaller than 5 cm. No significant differences were found in patients’ characteristics, median operative time, conversion rate, intraoperative hemodynamic and metabolic parameters, postoperative intensive care unit (ICU) admission rate, complications rate, and length of hospital stay. Long-term oncologic outcomes were similar, with a recurrence rate of 5.1% in group 1 vs 3.6% in group 2 (*p* = 1).

**Conclusion:**

Minimally invasive adrenalectomy seems to be safe and effective even in large PCC. The recommendation to prefer an open approach for large PCCs should probably be reconsidered.

## Introduction

Since its first description in 1992 [1], laparoscopic adrenalectomy (LA) has become the standard of care for the surgical treatment of most adrenal tumors. Compared with open adrenalectomy (OA), LA is reported to have better short-term outcomes, such as lower perioperative morbidity and faster postoperative recovery [2]. Pheochromocytoma (PCC) is a catecholamine-secreting tumor. It is a life-threatening condition due to the potentially lethal cardiovascular [3, 4] effects caused by catecholamine secretion. Cerebrovascular [5], renal, and gastrointestinal [6, 7] complications are also described. Surgical resection is the only definitive treatment for PCC. However, the laparoscopic approach can be technically challenging because of the unique biologic behavior of this tumor: PCC is a highly vascular tumor, tends to adhere to adjacent structures, and is usually larger than other adrenal lesions. Therefore, LA for PCC, even in experienced hands, may be associated with longer operative time and increased complications when compared with LA for other adrenal diseases [8–10].

Several risk factors for perioperative morbidity after LA have been identified [10–15], but only few studies have investigated specific predictors of morbidity after LA for PCC [16–20]. In particular, the role of PCC size, one of the most frequently reported predictor of perioperative complications, is controversial [21–23]. Guidelines on pheochromocytoma and paraganglioma published in 2014 by the Endocrine Society recommend open resection for large or invasive PCC to ensure complete tumor resection, prevent tumor rupture, and avoid local recurrence [24]. There are also concerns about the fact that, especially in large tumors, pneumoperitoneum could worsen intraoperative hyper and hypotensive peaks, making anesthetic management more challenging and risky. At our institution, a minimally invasive approach was attempted in all scheduled adrenalectomies, included adrenalectomies for PCC, irrespective of tumor size. The aim of this study was to analyze intraoperative and short-term outcomes of LA for PCC, comparing results in patients affected by lesions larger and smaller than 5 cm.

## Materials and methods

At our institution, laparoscopic adrenalectomy was introduced in the mid-1990s. After completing the surgical learning curve, a prospective protocol including all aspects of perioperative management of PCC was established, and all consecutive patients were included in a specific database. From July 1998 to June 2020, complete medical records of 114 consecutive patients who underwent a minimally invasive adrenalectomy for PCC were reviewed. The preoperative workup included clinical evaluation, biochemical tests (measurement of fractionated urinary/plasma catecholamines and metanephrines and serum chromogranin A levels), abdominal ultrasonography (US), and abdominal computed tomography (CT) to assess the size and the relationship with adjacent organs. Magnetic resonance imaging (MRI) and 123I-MIBG Scintigraphy or 18F-FDOPA PET were performed in selected cases with equivocal CT or discrepancy between imaging studies and biochemical tests. In addition, echocardiography was performed in all patients to rule out any underlying cardiac disease. Every case was discussed during our department’s weekly multi-disciplinary team (MDT) meeting.

All patients received a standard preoperative pharmacological treatment: selective alpha-adrenergic blocker (doxazosin) was administered at least 15 days before operation, and a beta-1 selective blocker was added if concomitant tachycardia was present. During the operation, alpha-adrenergic blockers (urapidil) and beta-blockers (labetalol) were used to manage intraoperative hypertension and sinus tachycardia, respectively. Intraoperative and postoperative hypotension was treated with volume expansion or, if necessary, with norepinephrine infusion. All operations were performed by two surgeons (G.G., M.M.) highly experienced in laparoscopic surgery. In 103 cases, LA was performed transperitoneally with the patient in lateral flank position as described previously [25], maintaining the intra-abdominal pressure at 12 mmHg. In 11 cases, a retroperitoneoscopic access was preferred, with a CO2 pressure of 20 mmHg. Direct manipulation of the tumor was avoided, and, in order to minimize the catecholamine release, a complete voiding of the adrenal lodge represented the surgical strategy. In case of bilateral lesions, two separate adrenalectomies were performed by turning the patient from the left to the right flank position during a unique anesthesia. Suction drains and nasogastric tubes were not routinely used. Postoperative monitoring was ensured in a surgical ward or, if suggested by the anesthesiologist, in an intensive care unit (ICU) for the first postoperative day; all patients underwent an endocrinologic evaluation before discharge.

Two groups were defined: patients affected by tumors larger than 5 cm and patients affected by tumors up to 5 cm. The following variables were recorded for each patient: age, sex, BMI, medical comorbidities, American Society of Anesthesiologists (ASA) score, fractionated urinary catecholamine and metanephrine levels, preoperative symptoms (presence at least of one of the following: headache, diaphoresis, palpitations, nausea, dizziness, asthenia, weight loss, anxiety, tremor, agitation, abdominal pain, fever), history of hypertension (defined as the assumption of at least one antihypertensive drug before diagnosis of PCC), resection margin involvement, biologically aggressive behavior (evaluated according to the Pheochromocytoma of the Adrenal Gland Scaled Score (PASS) [26]), operative time, need of intraoperative blood transfusion and drugs (both hypotensive and vasopressors), intraoperative tachycardia (frequency > 100 bpm), desaturation (SpO2 < 90%), acidosis (pH < 7.3) and hypoglycemia (< 70 mg/dl), intra- and postoperative hypertensive crisis (defined as a SBP higher or equal to 180 mmHg), intra- and postoperative hypotensive episodes (defined as a SBP lower or equal to 80 mmHg), intra- and postoperative morbidity (defined as any local or systemic complication occurring within 30 days of operation) according to Dindo’s classification [27], and hospital stay.

All patients signed an informed consent before the operation; this case series has been reported in line with the PROCESS Guideline [28]

### Statistical analysis

Continuous variables are reported as median and inter-quartile range (IQR) after the evaluation of normality with the Shapiro–Wilk test and categorical variables as events number and percentages. Differences between the 2 groups (defined considering the cut-off of 5 cm for lesion size) were tested with the chi-square test for categorical variables (with Fisher’s correction when needed) and with the Mann–Whitney test for continuous ones. All *p*-values are two sided, with a conventional level of significance of 5%.

## Results

During the study period, 114 patients underwent 118 adrenalectomies for PCC. A laparoscopic approach was attempted in all cases. LAs for lesions larger than 5 cm (group 1) were performed in 46 patients (40.4%), while for tumors smaller than 5 cm (group 2) in 68 patients (59.7%). In this group, a retroperitoneal approach was chosen on 11 cases; the relative low number is due to the fact that retroperitoneal technique was only recently adopted at our center. Median tumor size was 6.0 cm in group 1 (range 5.1–15.0) and 3.0 cm in group 2 (range 1.2–4.9). No significant differences were found in patients characteristics except a higher rate of preoperative symptoms in group 1, as summarized in Table 1. Four transperitoneal LAs were performed by means of robotic assistance (Da Vinci Si); 2 of them were larger than 5 cm. In 3 patients that were not able to tolerate pneumoperitoneum at a standard pressure because of severe cardiopulmonary and cerebral comorbidities, a gasless laparoscopic adrenalectomy with abdominal wall lifting was performed.

**Table 1:** Comparison between > 5 and ≤ 5 cm PCC: pre-operative data

	Pheochromocytoma				
	> 5 cm		≤ 5 cm		*p*-value
Operations (*n*; %)	46	100%	68	100%	
Female sex	20	43.5%	40	58.8%	0.107^*^
Age [years] (median; range)	48(15–77)		53 (17–84)		0.168^#^
BMI [Kg/m^2^] (median; range)	24.0 (16.4–32.8)		24.3 (16.0–41.5)		0.596^#^
Medical morbidities (*n*; %)					0.641^*^
Vascular	8	17.4%	13	19.1%	0.756^*^
Pneumologic	14	30.4%	25	36.8%	0.485^*^
Cardiac	9	19.6%	22	32.4%	0.132^*^
Kidney	8	17.4%	12	17.7%	0.972^*^
Gastrointestinal	12	26.1%	22	32.4%	0.473^*^
Neurologic	8	17.4%	12	17.7%	0.972^*^
ASA score (median; range)	3 (1–3)		2 (1–4)		0.424^#^
Pre-op hypertension (*n*; %)	39	84.8%	52	76.5%	0.278^*^
Pre-op symptoms (*n*; %)	29	63.0%	24	35.3	0.004^*^
Pre-op elevated urinary metanephrine levels	42	91.3%	54	81.8%	0.118^F^
Postoperative ICU (*n*; %)	14	30.4%	12	17.7%	0.110^*^

^*^Chi square test

^#^Mann–Whitney test

^F^Fisher’s exact test

Median operative time was 120 min in group 1 vs 105 min in group 2 (*p* = 0.395).

There were 3 cases of intraoperative bleeding who required intraoperative blood transfusion, two of which in the large tumors group. In one case from the first group, the intraoperative blood loss was secondary to the presence of adrenal vein neoplastic thrombosis that required conversion to open surgery, thrombectomy, and inferior vena cava repair by hand-sewn running suture; in the other two cases, the intraoperative bleeding was secondary to difficult dissection maneuvers.

Conversion rate was 4.3% (2/46) in large tumors group and 0% in small tumors group (*p* = 0.169). In both cases, conversion was necessary because of locally advanced right adrenal tumors adherent to the inferior vena cava. No significant differences were recorded between the two groups among all the intraoperative analyzed data (Table 2). Of note, intraoperative hypertensive peaks and hypotensive episodes showed a similar occurrence rate in the two groups.

**Table 2:** Comparison between > 5 and ≤ 5 cm PCC: intraoperative data

	Pheochromocytoma				
	> 5 cm		≤ 5 cm		*p*-value
*N*	46		68		
Operative time [min] (median; range)	120 (60–340)		105 (50–360)		0.379^#^
Conversion rate	2	4.4%	0		0.169^F^
Intra-op blood transfusion (*n*; %)	2	4.4%	1	1.5%	0.564^F^
Intra-op hypotensive drugs (*n*; %)	39	84.8%	50	73.5%	0.154^*^
Intra-op vasopressors (*n*; %)	4	8.9%	5	7.4%	0.521^F^
Intra-op tachycardia	9	19.6%	8	11.8%	0.251^*^
Intra-op desaturation	1	2.2%	1	1.5%	1^F^
Intra-op hypertensive peaks	31	69.6%	45	66.2%	0.705^*^
Intra-op hypotension	12	26.1%	17	25.0%	0.896^*^
Intra-op acidosis	9	19.6%	11	16.2%	0.641^*^
Intra-op hypoglycemia	0		1	1.5%	0.409^*^

^*^Chi square test

^F^Fisher’s exact test

^#^Mann–Whitney test

Postoperative ICU admission rate was 30.4% (14/46) in the large tumors group and 17.7% (12/68) in the small tumors group (*p* = 0.110); postoperative complication rate and length of hospital stay were comparable (Table 3).

**Table 3:** Comparison between > 5 and ≤ 5 cm PCC: postoperative data

	Pheochromocytoma					
	> 5 cm		≤ 5 cm			p-value
*N*	46		68			
Post-op ICU admission rate	14	30.4%	12		17.7%	0.110^*^
Length of hospital stay [days] (median; range)	5 (2–21)		4 (2–37)			0.453^#^
Complications (*n*; %) within 30 days	16	34.8%	17	25.0%		0.259^*^
Dindo 1–2	12	26.1%	13	19.1%		0.378^*^
Dindo 3–4	4	8.7%	4	5.9%		0.713^F^
Dindo 5	0		0			
PASS score ≥ 6	7/24	29.2%	10/34	29.4%		0.984^*^

^*^Chi square test

^F^Fisher’s exact test

^#^Mann–Whitney test

Postoperative morbidity was similar: in group one, two patients were admitted in the ICU for acute respiratory failure; one patient underwent a pace-maker implantation for an atrioventricular block, and a surgical revision for intestinal occlusion was necessary in the fourth patient. In group two, a bronchoscopic management of central airway obstruction was needed in two cases; one patient was admitted to the ICU for a myocardial infarction, and a percutaneous tube was inserted to drain an abdominal fluid collection in the last case. None of the patients died within 30 days from the operation.

A negative resection margin was confirmed by the pathologist in 45/46 (99.2%) specimens in group 1 and in all the specimens in group 2. No significant differences were found between the 2 groups in terms of PASS score choosing a cut-off value of 6 as predictor of aggressive behavior (Table 4).

**Table 4:** Oncologic outcome

	Pheochromocytoma	
	> 5 cm	≤ 5 cm
*N*	46	68
Tumor size [cm] (median; range)	6 (5.1–15)	3 (1.2–4.9)
Negative resection margin	45 (97.8%)	68 (100%)
PASS Score (median; range)	3 (0–9)	4 (0–10)
Genetic syndrome		
MEN 2a	0	2
MEN 2b	1	2
Von Hippel Lindau	1	0
Neurofibromatosis 1	1	1
FU [months] (median; range)	70 (6–160)	
FU rate	39/46 (84.8%)	56/68 (82.4%)
Local recurrence rate	2/39 (5.1%)	2/56 (3.6%)
Contralateral side	1/2	2/2
Distant metastases	1/39 (2.6%)	0/56

Follow-up rate was 83.3% (95/114), with a median of 70 months (6–160).A total of 4/95 (4.2%) recurrent lesions was recorded, 2/39 (5.1%) in group 1 and 2/56 in group 2 (3.6%); *p* = 1. A PASS score > 6 was defined on pathological report in all of these specimens. One of these patients developed an early recurrence on the same side with multiple distant metastasis. He was not reoperated and died for disseminated disease. The other three patients, affected by MEN 2A, MEN 2B, and von Hippel Lindau syndrome, respectively, developed a pheochromocytoma on the other side and were reoperated with a laparoscopic approach. One patient is free from disease at 6 years; the other two died for metastases of GIST and breast cancer, not related to the adrenal tumor.

## Discussion

PCCs are rare neuroendocrine tumors associated with high morbidity and mortality rate, due to cardiovascular complications and to potential tumor recurrence [29]. Laparoscopy has reduced perioperative morbidity when compared with OA, becoming the treatment of choice for PCC [2, 30–33]. Nevertheless, LA for PCC is still generally associated with longer operative time, higher conversion and complication rates compared with LA for different indications [8–10, 12], and data about specific perioperative predictors of outcomes in this population which are limited in the literature.

Surgical manipulation, intubation, and also the induction of pneumoperitoneum have been shown to cause a massive release of catecholamine during LA, resulting in severe hypertension and cardiovascular instability [33, 34]. A study from the Netherlands [35] showed how, in patients who are operated for a PCC, high preoperative plasma norepinephrine levels, tumor size larger than 4 cm, high blood pressure at presentation and after adrenergic alfa-receptor blockade, and a more pronounced postural drop (> 10 mmHg) in blood pressure after alfa-receptor blockade predispose to intraoperative hypertension. Urinary epinephrine and norepinephrine levels and tumor size have been identified as risk factors for intraoperative hypertension and postoperative hypotension in other more recent papers [36–39]. It is still a matter of debate whether LA for large adrenal tumors is safe and feasible. Several studies show that, especially in case of PCC, large adrenal lesions are associated with higher rates of conversion and blood loss, longer operative time, and hospital stay [10–12, 15–20], while others failed to find a correlation between tumor size and perioperative outcomes [21–23]. Laparoscopic approach was not identified as a variable influencing perioperative outcomes in recent studies [36–39]; furthermore, Kiernan et al. [40] reported how the open technique significantly increased intra- and postoperative hemodynamic instability. Our study shows that tumor size does not influence perioperative hemodynamic instability. No significant differences were found in intraoperative hemodynamic, respiratory, and metabolic parameters analyzed between the two groups. Furthermore, conversion rate was comparable, and a higher postoperative complication rate in patients with lesions larger than 5 cm did not occur in our series. These results are in line with a recently published meta-analysis [41] including 743 patients from 13 retrospective studies and one RCT comparing laparoscopic vs open adrenalectomies for PCC. Authors concluded that laparoscopic adrenalectomy shows lower estimated blood loss, lower transfusion rate, lower hemodynamic instability, less postoperative complications, less Clavien-Dindo score 3 complications, shorter return to diet time, and shorter length of hospital stay. Nevertheless laparoscopic group might have smaller tumor size but higher body mass index. The tumor size issue (about 1 cm difference between the two groups) could be due to the inherent selection bias in which tumors are amenable to laparoscopic approach, especially in older series, and its influence on surgical outcome is of little significance. There are no data reporting differences in recurrence rate between open and laparoscopic approach. Goffredo et al. [42] reported a series of 287 malignant PCC, being 72.5% larger than 5 cm. Tumor recurrence or persistence was present in the 3–13% of the PCC operated, and 5-year overall survival was significantly higher for patients with tumors measuring less than 5 cm (72.5% vs 27.5%). In our series, with a 16.7% rate of patients lost at follow-up, a recurrence rate of 3.2% was registered, with an overall survival rate of 96.8%. This can be explained by the fact that our series includes all PCC, both benign and malignant, but tumor size seems not to influence long-term outcomes (5.1% vs 1.8%). At our center, a minimally invasive approach was attempted in all cases, irrespective of tumor size. Nevertheless, a radical resection performed maintaining a good hemodynamic control must be the final goal, and surgeons have to be ready to convert to open surgery whenever the situation requires it. In our series, we reported only one case of conversion in which unfortunately, despite the open technique, the resection was not radical and the patient developed an early recurrence associated to disease dissemination which died for.

This study is subject to the inherent limitations of any retrospective study, such as selective reporting and incomplete data bias. Nevertheless we made great efforts to overcome these limitations with a thorough review of medical and anesthetic charts, in order to record a great number of parameter to include in the analysis. It is important to note that this is a consecutive series including all PCC surgically treated during the study period and managed with a standard perioperative prospective protocol.

## Conclusion

Minimally invasive adrenalectomy represents the treatment of choice of PCC. Even in tumors larger than 5 cm, laparoscopic approach does not affect intraoperative and short-term outcomes and does not worsen the hemodynamic instability typical of this disease. Recurrence rate and overall survival rate are comparable to the reported series of open resections, irrespective of tumor size.

The recommendation to prefer an open approach for PCCs larger than 6 cm [24] is not supported by strong evidence and, considering the benefits and the results of a minimally invasive approach, should probably be reconsidered.

## Data Availability

The datasets generated and analyzed during the current study are available from the corresponding author on reasonable request.
